# MCDA-based deliberation to value health states: lessons learned from a pilot study

**DOI:** 10.1186/s12955-019-1189-7

**Published:** 2019-07-01

**Authors:** Fabia Gansen, Julian Klinger, Wolf Rogowski

**Affiliations:** Department of Health Care Management, Institute of Public Health and Nursing Research, Health Sciences, University of Bremen, Bremen, Germany

**Keywords:** Health state valuation, SF-6D, Deliberation, MCDA, MACBETH, Germany

## Abstract

**Background:**

Health economists have shown a growing interest in deliberation and multi-criteria decision analysis (MCDA) as possible pathways to transparently integrate value judgments in cost-utility analyses. In line with these developments, this study piloted a consensus process to derive a German value set for the Short-Form Six-Dimension (SF-6D). In a conference setting, a group was tasked to deliberate on scores and weights for the SF-6D from the perspective of a self-determined and independent life.

**Methods:**

The one-day consensus conference was based on a deliberative process in combination with the MCDA method MACBETH (Measuring Attractiveness by a Categorical Based Evaluation Technique). According to MACBETH, participants were asked to qualitatively rate pairwise comparisons of SF-6D health states. The scoring within each dimension was conducted in parallel group sessions. Final agreement on the scores as well as weights for the SF-6D dimensions were derived in a subsequent plenary assembly. Results were analyzed using the software M-MACBETH and qualitative content analysis.

**Results:**

A total of 34 participants were recruited. While each of the 6 small groups presented a consented score, the plenary assembly reached consensus on all dimensions apart from pain. Concerning dimension weights, some participants favored prioritizing pain and mental health. Others disputed that trade-offs between dimensions and thus assigning weights were not acceptable in a context where this may involve withholding care from someone. As a consequence, no consensus on a value set was reached. Participants identified the group size of the plenary session and the applied weighting procedure as main obstacles to the process.

**Conclusions:**

This pilot study presents a consensus-based approach for valuing health-related quality of life. However, further research is needed on deliberative processes that yield quantifiable results. Future conferences should explore smaller group sizes, longer durations of the deliberative process and alternatives to the additive value function applied in MACBETH.

**Electronic supplementary material:**

The online version of this article (10.1186/s12955-019-1189-7) contains supplementary material, which is available to authorized users.

## Background

In health economics, there has been a growing interest in the application of both multi-criteria decision analysis (MCDA) and deliberation [1, 2]. MCDA is an umbrella term for different approaches to assist decision makers in decisions with multiple objectives. Within health sciences, MCDA has been increasingly applied to fields such as medical decision making and the evaluation of healthcare interventions in the context of health technology assessment [3, 4]. Deliberation can be understood as a form of group discussion where participants debate public matters in an informed and considered manner. In democratic decision making and priority setting, deliberative procedures have been used to determine consensus views within a group [5]. In combination, the two approaches MCDA and deliberation may serve as a pathway to transparently integrate value judgments in cost-utility analyses. Against this background, this study presents a novel approach for applying MCDA and deliberation to examine the feasibility of deriving a consented value set the SF-6D or Short-Form Six-Dimension, an instrument to measure health-related quality of life (HRQOL).

Value sets for HRQOL instruments build the basis for measuring effects in cost-utility analyses of healthcare interventions. By providing health-state utility values, they enable the calculation of quality-adjusted life-years (QALYs). The QALY combines life years spent in a certain health state with the quality of this health state. This is done by giving a year of full health a value of 1, health states considered to be equivalent to death the value 0, and health states regarded as worse than death negative values [6]. Within health economic evaluations, the health state utility values can therewith capture differences in HRQOL and QALY corresponding to the interventions investigated. As such, the values assigned to HRQOL can have a substantial influence of the results of cost-utility analyses.

In the development of value sets for HRQOL, there has been a rising concern that instant responses to preference elicitation surveys can lead to unreasoned and inconsistent judgment decisions [5]. This ongoing debate has been fostered by reports of logical inconsistencies and connected quality issues in existing valuation data [7]. Hausman [8] claims that preferences can only provide a suitable basis for valuing health states if they satisfy the criteria of self-interest, rational deliberation and complete knowledge. According to Hausman, this is not the case in the surveys currently used for health state valuation [8]. As an alternative, he therefore proposes valuing health states within deliberative groups [8]. More fundamentally, in countries like Germany, there are both ethical concerns and political resistance against using methods of health economic evaluation for coverage decisions. In part, this is due to the fact that consequentialist principles like health maximization conflict with the deontological fairness considerations which guide constitutional and social law. Recently, it has been argued that these concerns could be addressed by analyzing health economic evaluation as a tool for conflict resolution rather than as a tool for maximizing any unit of outcome like health [9]. To further develop a methodology of health economic evaluation which is consistent with this theoretical framework, there is a need that all methodological elements can find consensus among those who are affected by coverage decisions.

To address these issues, this study explores a deliberative valuation of health-related quality of life based on consensus. This deliberation is conducted from a public perspective to enable participants to find common ground and comprehensible reasons for their judgment decisions. As such, this study explores one possible implementation for what Hausman calls eliciting public values for health states [8], adapted for the SF-6D. In line with Hausman’s concept of Public Value, the piloted framework aims at eliciting well-considered value judgments from a public perspective which – if possible – ultimately result in a consented value set for HRQOL.

Up to date, preference elicitation and value sets for HRQOL are generally based on large-scale surveys of the population. Applied techniques for eliciting individual’s preferences with regard to health state valuation vary and include time trade-off, standard gamble and discrete choice experiments [10]. Population-based surveys of individuals are used to estimate mean values for country-specific value sets. Traditionally, these value sets are generated using either statistical modelling or multi-attribute utility theory (MAUT). Applying statistical models to estimate value sets is also known as the composite approach. These econometric methods have been applied to value well-known HRQOL instruments such as the EQ-5D and SF-6D. The decomposed approach or MAUT in which the form of the utility function is specified before surveying has been less commonly used in the estimation of value sets [10, 11].

Given the above-mentioned critique [5, 8], the valuation approach implemented in this pilot study provides an alternative valuation method based on multi-attribute utility theory and deliberation. The combination of MCDA and public deliberation offers an apt means to facilitate and structure the valuation process and thus transparently assess value decisions. This investigation is in line with existing healthcare research on supporting deliberation with MCDA [12], the application of deliberation in the context of health state valuation [5, 13] and the use of MCDA techniques for valuing health [14]. To our knowledge, these methods have not yet been combined to value health from a public perspective in a group setting.

This study’s first implementation of the proposed consensus-based valuation method was conducted on the HRQOL instrument SF-6D. It was chosen for the purpose of this study since no value set for the German population has been published for this instrument yet. In a conference setting, a group was tasked to deliberate on scores and weights of the SF-6D from a public perspective. Thus, participants were asked to rate differences in health states from the perspective of an individual’s opportunity to lead a self-determined and independent life. Scoring and weighting were based on the MCDA method MACBETH – Measuring Attractiveness by a Categorical Based Evaluation Technique [15].

The research objective of this pilot study was to test whether or not the developed methodology is suited for citizens to reach consensus on a value set for a health-related quality of life (HRQOL) measure. This pilot study is meant to serve as a starting point for further advances in this research area and provide a methodology to develop consented value sets for HRQOL instruments as a basis for cost-utility analysis.

## Methods

### Methodological framework

#### Health-related quality of life

The SF-6D or Short-Form Six-Dimension is an established instrument for measuring health-related quality of life [10]. It is a generic preference-based measure which makes use of six dimensions to describe health states. These dimensions are physical functioning, role limitation, social functioning, pain, mental health and vitality. As is standard for generic health measures, the descriptive data defining each of these dimensions on different levels were obtained from patients. Previously developed value sets were typically obtained from the general public [6]. While the SF-6D is one of the most commonly used instruments to measure health outcomes, a value set for the German population has yet to be published.

The SF-6D is one of several instruments that have been developed to provide health state utility values. Other generic preference-based measures of health include the EQ-5D, Health Utilities Index (HUI), the Quality of Well-Being (QWB) scale, the 15D and the assessment of quality of life (AQoL) [10]. The SF-6D is derived from the SF-36 and its shortened version SF-12 which are generic health surveys consisting of multi-level items across 8 health dimensions [16]. Its development allows the conversion of data collected using the SF-36 or SF-12 to SF-6D health states. As a consequence, the SF-6D is not a questionnaire in itself which is issued to patients in clinical studies but an instrument developed specifically for use in health economic evaluations. For the purpose of this pilot study, the SF-6D Version 2 was applied [10]. To generate a complete descriptive system for the SF-6D Version 2 in German, the corresponding questions in the German versions of the SF-12 and SF-36 were utilized. The applied version of the SF-6D is available from the authors upon demand.

#### MCDA

To derive a value set for the SF-6D, this study applied a method of multi-criteria decision analysis or aiding (MCDA). In short, the term MCDA refers to “a collection of formal approaches which seek to take explicit account of multiple criteria in helping individuals or groups explore decisions that matter” [17]. MCDA is meant to provide a systematic and transparent procedure to take into account all relevant criteria and help prevent arbitrary misjudgments due to inconsistency [12, 18]. A variety of different MCDA approaches exists. They range from value measurement methods such as the MAUT-based method implemented in this study to outranking and goal programming [19]. For consistency with health valuation literature, MAUT is used here as a synonym for multi-attribute value theory or MAVT. The simplified assumptions made in MAUT about the form of the value function have the advantage of reducing the valuation task [10]. Compared to statistical modelling, only a relatively small sample of valuation decisions is needed to specify the value function. In the pilot study, this was done by conducting two steps present in many applications of MCDA: a scoring procedure to generate a partial value function and scale for each dimension as well as a weighting procedure to derive dimension weights. These scores and weights are inserted into the value function to derive a value for all 18,750 health states within the descriptive system of the SF-6D.

The MCDA method selected for this analysis is Measuring Attractiveness by a Categorical Based Evaluation Technique or MACBETH [15]. In contrast to many traditional health valuation and MCDA techniques, this method makes use of only qualitative judgments. It thereby eliminates the need for quantitative assessments. On the basis of pairwise comparisons, the MACBETH procedure derives numerical scales based on linear programming. To determine an overall value, MACBETH adopts an additive aggregation model. This simplification enabled a comprehensive and transparent value calculation. Besides the benefits associated to MAUT in general, MACBETH allowed for conducting the scoring and weighting procedure without the need for assessing specific options or alternatives. As the evaluation of a given set of alternatives is the aim of most MCDA applications, the methodology is generally constructed accordingly. MACBETH has been applied successfully both in healthcare [20] and group decision-making [21]. However, its application in healthcare is less common than methods such as the Analytic Hierarchy Process (AHP) [4]. Similar to MACBETH, AHP also uses pairwise comparisons but rates differences on a scale from 9 to 1 to 9 [22]. AHP is not based on MAUT [23] and uses the eigenvalue method instead of linear programming to calculate overall values [24].

For the scoring procedure, participants were asked to rate the difference in attractiveness of two health states which differed in the level of the dimension in question. The difference was assessed on a qualitative scale specific to the MACBETH method. This scale included the levels no difference, very weak, weak, moderate, strong, very strong and extreme difference and was used for both scoring and weighting. The weighting procedure followed Bana e Costa et al. [15]. It was to be conducted in two steps and set the worst health state of the SF-6D as a starting point. First, participants were asked to qualitatively value a change from the worst to the best level with the other dimensions remaining at the worst level for each dimension at a time. The second step contained a pairwise comparison of this change from the worst to best level between two dimensions.

With the elicited scores and weights, the value for a given SF-6D state could be calculated by multiplying each dimension weight with the score associated to this dimension’s level and adding up these products across all dimensions. The MACBETH technique was implemented by use of the software M-MACBETH in the version 2.5.0. To support the decision making of the participants, the software also gave feedback on inconsistencies and provided suggestions to resolve them.

#### Deliberation

While there has been a growing interest in public deliberation in the health sector [25], the term can be challenging to define [26, 27]. The goal of deliberation can be described as achieving informed and considered decisions [5, 28]. For the purpose of this study, a minimal definition of deliberation following Blacksher et al. [29] and findings from a review by Abelson et al. [25] was used. Thus, the process of deliberation was designed on the basis of three criteria: (1) the provision of balanced, factual information, (2) the inclusion of diverse perspectives and (3) the opportunity to reflect and discuss freely. Factual information was provided by informative material provided before and during the conference, an expert-led introductory presentation and a question and answer (Q&A) session. The informative material was concise and consisted of a few pages to avoid asking too much preparation of the participants. While diverse perspectives were encouraged, achieving representativeness was beyond the scope of this pilot study. The process was accompanied by neutral moderators to ensure participation to be free and equally considered.

The deliberative process of this study was designed with the goal of reaching a consensus within the group. Nevertheless, the possibility of not achieving an agreement was also communicated to the participants as a potential outcome of the study. To enable finding a consensus, participants were asked to take a public viewpoint with regard to their judgment decisions. In order to facilitate the utilization of public reasons, all valuing questions asked during the scoring and weighting procedures were ended with “from the perspective of leading a self-determined and independent life”. This phrase was meant to operationalize the Public Value concept by Hausman [8].

### Study design

#### Sample and setting

In view of the pilot character of this study, a convenience sample of university students was chosen for participation. Recruitment was thus conducted via e-mail to students of the Bachelor’s and Master’s Programs of Public Health and the Master’s Program Professional Public Decision Making at the University of Bremen. Additionally, students were personally approached in November and December 2017. The ethics committee of the University of Bremen waived the need for an ethics approval. All participants gave an informed and written consent for participation in and audio recordings during the conference.

The conference took place on December 15, 2017 between 9.15 a.m. and 5 p.m. in parallel small groups and subsequent plenary sessions. The one-day design of the conference was chosen to prevent excessive demand and limit participants’ expenditure of time. Additionally, semi-structured interviews with the moderators and software operators of each small group as well as with the operator of the weighting procedure were conducted on January 29, 2018. The topics of the interviews were a summary of the reasoning behind the group decisions, the scoring procedure and deliberation both in the small groups and in the plenary assembly as well as perceived difficulties. Corresponding questions were asked for the weighting procedure. The interviews were conducted by the researchers and authors of this study (FG, JK, WR). Each researcher conducted two interviews. A translated version of the interview guidelines originally developed and used in German can be found in Additional file 1.

#### Conference procedure

The conference was structured in four main sessions. It began with an introduction into the topic held by the senior researcher involved in the study (WR), followed by a presentation of the day’s schedule and a Q&A session. Next, the participants were divided into pre-defined small groups. Each of these groups was tasked with the scoring of one of the SF-6D dimensions. In the following, these groups are referred to as expert groups. After the expert group sessions, the plenary assembly – consisting of all participants – gathered for presentation and validation of the expert group results. Finally, the weighting procedure was conducted in the plenary assembly after an introduction by the session’s operator. Results were exemplified with selected case vignettes.

Selected Master’s students of Professional Public Decision Making moderated the discussion groups and operated the M-MACBETH software. These students were involved in the procedural development of the pilot study. They were supported by guidelines for moderation as well as a 1-day preparatory training. Beside the support provided during the deliberation by the moderator, the M-MACBETH software and its operator, information material was made available to the participants before and during the conference. This material included background information sent to the participants via e-mail 2 weeks before the conference, an overview of the questions on hand during the scoring procedure, a printout of the SF-6D V2 and details on the case vignettes for the presentation of results.

After the conference, participants filled out an evaluative questionnaire with closed questions on self-reflection and their perception of the conference procedure. The questions of self-reflection were based on the Perspective Taking Scale [30]. The questionnaire also contained the option to write comments, questions or concerns about the study. It was handed out and collected as a printout immediately after the last plenary session and available online with a link provided via e-mail for a period of 4-weeks after the conference.

#### Analysis

The analysis of the conference implemented both quantitative and qualitative research methods. The sample was analyzed on the basis of information provided upon registration and a preliminary questionnaire on personal information of the participants. The main results of the pilot conference are presented separately for the two parts of the SF-6D valuation, i. e. the scoring and weighting procedure conducted during the consensus conference. Their description is based on the M-MACBETH results and field notes of the proceedings taken during the conference. Additionally, the conference results include a quantitative analysis of the evaluative questionnaire’s closed questions.

To evaluate the pilot study, the focus of its appraisal lies on its methodological and overall challenges. Apart from the quantitative results of the concluding questionnaire, the evaluation is conducted primarily on the basis of a qualitative content analysis (QCA). The participants’ reasons for their judgment decisions were also analyzed as part of the QCA but are beyond the scope of this study. The QCA is based on the plenary sessions, the interviews of the moderators and software operators as well as the participants’ comments in the evaluative questionnaire. Field notes taken during the conference and after the interviews were not included in the QCA. The methodology of the QCA follows Schreier [31]. It was implemented in MAXQDA 2018, a software package for qualitative methods research.

For the purpose of the qualitative investigation, the plenary sessions and interviews were audio recorded. These recordings were transcribed verbatim using a defined standard [32]. The coding framework was primarily data-driven and developed inductively (by FG) using the method of subsumption. It was implemented by two coders (FG and JK) in a pilot phase. Development and piloting was based on an overlapping sample of the text material. After calculating the text-specific and overall inter-rater reliability in percentage of agreement the coding frame was revised. Following this, the frame was evaluated with regard to its feasibility and tested by two additional researchers not involved in its development. Both the pilot phase and the final coding of the full text material was conducted by two coders (FG and JK). The percentages of agreement were determined and differences in coding present after the final coding were discussed and resolved unanimously. Results of the QCA are presented with regard to evaluation-related categories on procedure, methodology and research approach, supplemented by expressed suggestions for improvement. Findings are illustrated by quotes translated from original statements made in German to English. Ellipses indicate missing speech.

## Results

### Sample and proceedings

A total number of 34 students participated in the conference. One registered participant did not attend. The sample mainly included Public Health students completing the Bachelor’s program at the University of Bremen (*n* = 24). Additionally, students in the Master’s Program Public Health (*n* = 5) and Professional Public Decision Making (*n* = 5) took part. The age of the participants was between 19 and 53 with a mean age of 25. Additional information on the conference participants according to the preliminary questionnaire can be found in Table 1.

**Table 1:** Main characteristics of consensus conference participants

	Number	Percent
Age		
Under 20	2	6%
Between 20 and 24	16	47%
Between 25 and 29	13	38%
At least 30	3	9%
Gender		
Female	29	85%
Male	4	12%
Not specified	1	3%
Residence		
Bremen	22	65%
Verden	2	6%
Other	10	29%
Qualification		
(Fach-)Abitur^a^	20	59%
University degree	12	35%
Other	2	6%
Healthcare Professional		
No	22	65%
Yes	12	35%

^a^German higher education entrance qualification

Every expert group during the scoring sessions consisted of 5 or 6 participants. Allocation to the expert groups was pre-decided and based on gender and study program to allow diversity within groups to a certain extent. Deliberation within the expert groups took between 45 min and 1 h and 37 min with a mean duration of 1 h and 16 min. The plenary sessions took 1 h and 45 min for scoring and 1 h and 11 min for weighting. With regard to the interviews conducted after the conference, 9 of the 12 moderators and operators took part. The duration of the interviews was between 17 and 40 min with an average time of 28 min.

### Conference results

#### SF-6D valuation

**Fig. 1:**
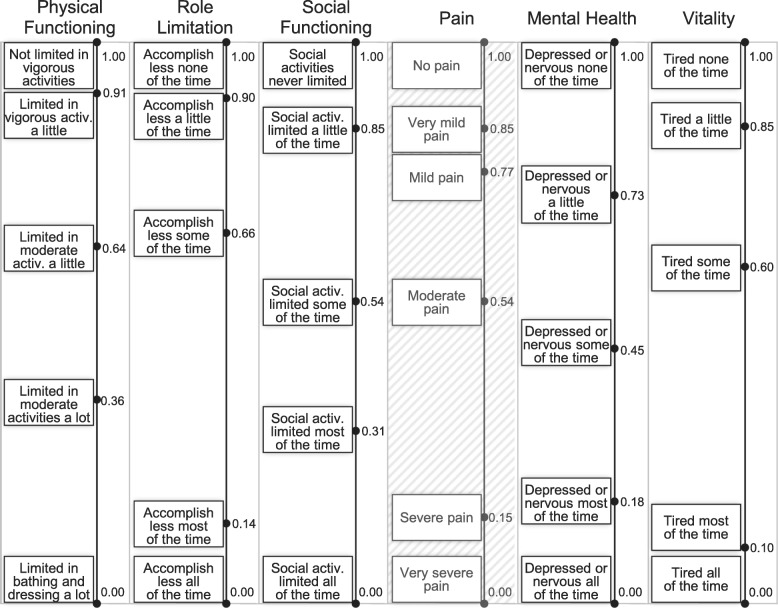
Results of the scoring sessions for the SF-6D dimensions. The presented numbers indicate the score of each level on a scale from 0 to 1. The scales in the dimensions physical functioning, role limitation, social functioning, mental health and vitality were agreed upon in the plenary assembly. The illustrated scale in the dimension pain was developed in the expert group but not consented by the plenary assembly. The abbreviation activ. stands for activities

Within the expert groups, consensus was reached for each of the 6 dimensions of the SF-6D. In the plenary session on scoring, consensus was achieved on 5 out of 6 dimensions. Ultimately, the plenary assembly decided not to change any of the expert group results. The final scales for the SF-6D dimensions are displayed in Fig. 1. For the dimension pain, no consented scale was decided on by the plenary assembly. The disagreement was due to a lacking consensus on the difference in attractiveness between the levels no pain and very mild pain. While some participants suggested increasing the difference between the two levels by lowering the partial utility value of very mild pain, others made the case for not adjusting the scale. As no final result was supported by the plenary assembly, a consented scale for the dimension pain was not concluded.

**Fig. 2:**
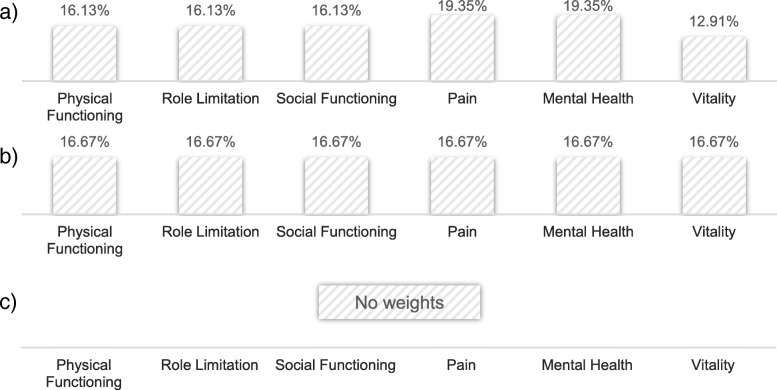
Non-consented results of the weighting procedure for the SF-6D dimensions. The presented numbers indicate the weight of each dimension. **a**) Result of prioritizing pain and mental health while giving vitality a smaller weight, **b**) All dimensions are given the same weight and **c**) No deduction of weights for the SF-6D dimensions. The striped illustration indicates that no consensus was reached on the results

The weighting session led to no final consented results for dimension weights. In summary, the arguments expressed by individual participants can be grouped to three diverse points of view. One group of participants was in favor of prioritizing the dimensions pain and mental health and saw a change in the dimension vitality as less impactful. Other participants expressed concerns about making trade-offs between the dimensions of health-related quality of life represented in the SF-6D. Some representatives of this standpoint argued the case for equal weights for all dimensions. A third group was not willing to accept trade-offs. These participants advocated not performing a weighting procedure primarily referring to ethical concerns. While the plenary assembly decided to forgo the second part of the weighting procedure, weights for two of these three positions could be derived. The results are illustrated in Fig. 2. In conclusion, no consented value set was developed by the plenary assembly.

#### Questionnaire

**Fig. 3:**
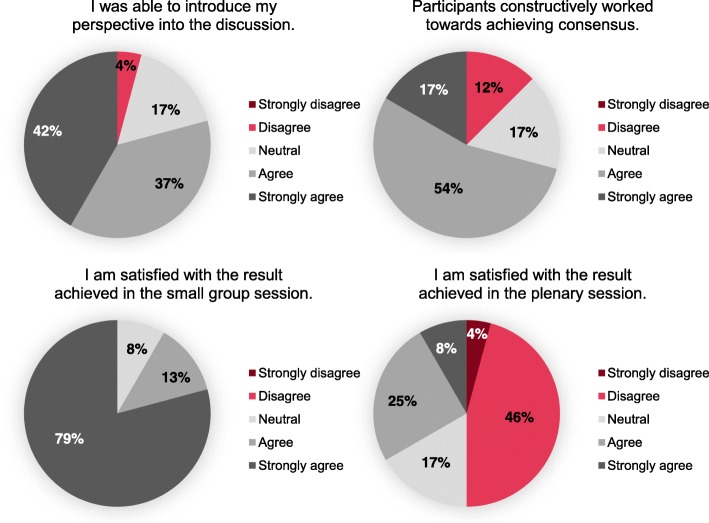
Selected results of the evaluative questionnaire’s closed questions

A total number of 24 participants filled in the concluding questionnaire. Selected results of the participant’s answers to its closed questions can be found in Fig. 3. Overall, participants tended to be satisfied with the results of the expert groups but less with those of the plenary assembly. Most participants agreed or strongly agreed that they were able to introduce their perspective into the discussion and the group constructively worked towards achieving consensus. Additionally, a majority agreed that participants responded to statements of others and disagreed that interruptions by other participants occurred often. Finally, participants predominantly felt that the information provided was adequate and that there was sufficient room for questions. The answers to the self-reflective questions on the Perspective Taking Scale showed that most participants viewed themselves as open to considering other perspectives. A detailed overview on the results of the questionnaire is given in Additional file 2.

### QCA-based evaluation

Several matters of context have to be noted regarding the QCA-based evaluation. One of these is the nature of the text material analyzed. Participants’ remarks from the plenary assembly were made while deliberating on scores and weights not specifically to evaluate the study design. Nevertheless, participants addressed issues they saw with regard to the methodology since the present researchers openly communicated the pilot character of the study. As a consequence, their remarks were analyzed with regard to their evaluative features and complemented by the comments in the questionnaires and interviews. Additional matters of context are the setting and participants of the conference. First, only students predominantly with a background in health sciences participated in the study. This limits the transferability of the findings presented. Second, most participants knew the researchers as lecturers in university courses. While participation in the consensus conference was not graded or directly linked to classes, the previously established relationship between participants and researchers may have influenced results. To reduce possible bias, the researchers of this study primarily had an observant capacity and intervened for support only when clarification was needed.

The complete coding frame developed for the QCA consisted of 9 main categories and 69 subcategories. The included categories and final number of codings are listed in Additional file 3. After the final coding and before resolving the divergence, the overall percentage of agreement between the two coders for all categories was 76.9%. The following evaluation is primarily based on the categories “Aspects of research approach evaluated”, “Aspects of methodology evaluated” and “Aspects of procedure evaluated”. The categories “Suggestions for improvement” and “Aspects of participants’ behavior evaluated” were also considered but are not reported separately. Consequently, the following findings of the QCA-based evaluation are presented in three sections. They begin with assessments of the overall research approach. These are followed by findings with regard to the methodology and its implementation in the consensus conference.

#### Research approach

With regard to the research approach presented in this pilot study, the main issues raised by participants were of ethical nature. These concerns were expressed primarily during the weighting procedure as several participants questioned the ethical acceptability of making trade-offs between dimensions. Some participants argued that it was intolerable to make judgment decisions when this could lead to withholding care from someone:

*“I find it even harder to decide when it’s ultimately about health insurances. So in my imagination it’s about deciding. I compare and have people sitting in front of me and then I have to say, well, yours is not too bad so your treatment won’t be paid for. But I think yours is worse so your treatment will be paid for. I think that is even worse, even more terrible to decide that because that’s just unacceptable.”* – Participant in weighting session.

Other statements about the research approach criticized its underlying assumptions, notable influences with impact on results and the overall complexity of the research question on hand. One participant remarked:

*“You can’t break down every question so simply. […] Many questions are so complex that you can’t answer them on the spot.”* – Participant in scoring session on social functioning.

Despite the problems identified related to the approach, individual participants also highlighted the practical relevance of the research question:

*“In practice we have 6 patients with 6 different extreme illnesses and have to decide whom to help first and whom to help next. […] As a healthcare system, we are in the position of […] having to make this decision in practice. And that has to be possible somehow according to certain criteria.”* – Participant in weighting session.

#### Methodology

With respect to the methodology applied in the consensus conference, participants, software operators and moderators identified several points to consider for further development. First of all, problems arose concerning definitions of words and phrases used in the SF-6D descriptive system. Both in the expert groups and the plenary assembly, questions on how certain terms are defined reoccurred. Many groups found their own definitions for the SF-6D levels e.g. what limited “a little”, “some” and “most” of the time meant as a basis for their discussion. The expert groups then introduced and clarified their understanding of their dimension to the plenary assembly. Similarly, participants felt that terms such as “depression” or “tired” needed defining before they could be discussed. The issue of unclear definitions is mirrored in a large number of remarks and codings in that regard. As one software operator pointed out, different understandings of terminology could lead to inconsistencies in judgment decisions:

*“I find that very problematic because in the other small groups other definitions were probably made and it is already inconsistent if you transfer it to the big matrix in the end. So one should have made a uniform definition.”* – Operator of the expert group on vitality in interview.

The understanding of the SF-6D descriptive system was complicated further by the wording of some dimensions. The expert group deliberating the dimension role limitation found the double negative used to describe the levels challenging when making judgments decisions (e.g. accomplish less none of the time).

Other issues brought up by participants concerned the assumptions made in the MCDA approach on preferential independence of and trade-offs between the SF-6D dimensions. These concerns presented themselves in the weighting session. Here, participants were asked to value an improvement in only one dimension from the worst to the best level with all other dimensions remaining the same. Several participants called into question the existence and therefore valuation of such a theoretical change in health:

*“I think it is hardly possible to move away from none of them influencing the others because they all have an effect on each other so it is not at all possible.”* – Participant in weighting session.

As described in the context of ethical concerns about the research approach, some participant refused to allow for trade-offs between the SF-6D dimensions:

*“I think you just can’t compensate one dimension with another.”* – Participant in weighting session.

Final methodological issues were connected to the understanding of the overall methodology and the meaning of finding a consensus. Despite many participants having a background in healthcare, it became clear that comprehending the method was challenging. In part, participants had difficulties in taking the public perspective and understanding the questions posed e.g. during the weighting procedure. Individual participants also made clear that on certain aspects they were unwilling to compromise ultimately preventing a consensus. Although information material and moderators pointed out that finding a consensus did not mean the result would reflect every participant’s personal opinion, individual comments indicated limited understanding and willingness to work towards consensus:

*“I believe it is simply impossible that we come to a consensus because if some people think that it is ethically forbidden to think about it at all then we can’t start a discussion about it and weigh the arguments.”* – Participant in weighting session.

#### Implementation

Procedural issues addressed by participants, moderators and operators can be summarized in aspects of organization, communication and tasks. Overall, participants were satisfied with the organizational implementation of the conference. Particularly the expert group sessions were evaluated positively. One of the main issues with regard to the organization of the consensus conference was the group size of the plenary assembly:

*“The second part of the event shouldn’t have taken place in a plenum since only few people took part in the discussion.”* – Comment of participant in questionnaire.

The time management of the 1-day conference was also addressed. Several participants remarked that they would have preferred more time for discussion. However, some participants also commented on the conference being too long. To improve future conferences, participants suggested longer time horizons e.g. of two days and smaller group sizes.

Another topic connected to implementation was communication. Respective comments addressed limited information and insecurity about the intended process or topic on hand. Suggestions for improvement included the integration of expert opinions. With regard to the information conveyed, one of the operators remarked:

*“Overall I didn’t feel like all information came across. Or it came across but was forgotten again quickly.”* – Operator of the expert group on vitality in interview.

Finally, participants had difficulties with some of the tasks they were given. Particularly at the end of the conference, participants expressed their exhaustion. Additionally, participants found certain assignments challenging. One of these was the final scoring procedure in the plenary session in which the results of the expert groups were validated by the plenary assembly. Several participants felt uncomfortable challenging scores which were extensively discussed by the expert groups:

*“Well, I just find it difficult, we’ve been concerned with our topic for so long and have discussed it for 1.5 hours. Thinking about the other items again is so far away now, I just can’t do that right now.”* – Participant in scoring session on social functioning.

*“Just from a headline to say now we’re changing what the others have discussed for such a long time. I don’t want to presume to want to change another group’s result.”* – Participant in scoring session on social functioning.

## Discussion

This study constitutes the first implementation of a deliberative valuation of HRQOL from a public perspective supported by MCDA. Particularly the consented dimension scales derived by the expert groups suggest that such an approach is feasible in principle. However, the pilot study did not result in a consented value set. In the weighting procedure, participants either opposed dimension weights altogether, advocated equal weights, or prioritized mental health and pain. While these results are preliminary, the last position is comparable to previous valuation studies of the SF-6D. Findings from the UK indicate that pain followed by mental health appear to be the most important dimensions in determining health state values [16, 33].

The context of the pilot study limits transferability of its results. Nevertheless, the implementation and its evaluation demonstrated a number of points to consider in future work on group deliberation and consensus for methods of health valuation and economic evaluation. In the following, these issues are discussed within the methodological framework of HRQOL, MCDA and deliberation.

### Health-related quality of life

One of the main issues identified with regard to the health-related quality of life measure chosen was the interpretation of SF-6D wording. While deciding on individual group interpretations was an obvious solution to the participants of the consensus conference, they expressed their concern about using incorrect definitions. Additionally, different interpretations could lead to inconsistencies especially when the result is composed of diverse understandings of the same phrases. Although controversial among participants, this issue was at least partially addressed by validating the expert group results in the plenary assembly.

Overall, it seems that the switch in perspective – from an individual to a bystander – poses challenges for the interpretation of descriptors. However, difficulties with wording and definitions could also be present in an individual perspective. In fact, diverse understandings and interpretations are likely present but not made explicit in any traditional HRQOL valuation study. In many cases, individuals are asked for their intuitive assessments and implicitly apply their understanding of the posed question without having to explain their decisions. The deliberative setting suggested by Hausman [8] could solve this previously disregarded issue of traditional valuation studies – provided the group can decide on a uniform understanding. This process could be supported by expert opinion or patient insights to help participants overcome insecurities about definitions. Nevertheless, the difficulty of finding generally valid definitions and subsequently making valuation decision about health from a public and not from a personal perspective could remain.

While the question of interpretation is likely to be present in any generic preference-based measure, it could be aggravated by the nature of the SF-6D. The SF-6D is not a questionnaire in itself but a shortened deviation of the SF-12 and SF-36 [16]. As a consequence, study participants had little additional information or assistance for interpretation provided in a questionnaire such as the 4-week time horizon given in the SF-36. By deliberating about the descriptive system of the SF-6D, participants also rated differences between health states not directly elicited by the original questionnaires. In conclusion, other generic preference-based measure of health-related quality of life may be more valid in a deliberative valuation approach.

### MCDA

Although ultimately the MCDA approach applied in the consensus conference did not result in a consented value set, it provided a transparent procedure and clear structure for deliberation. Furthermore, MACBETH avoided quantitative ratings and inconsistent judgments decisions. Although the phrasing of the weighting questions proved difficult, participants demonstrated better understanding than expected. Questioning could further be improved by deviating from the traditional MACBETH wording asking for “difference in attractiveness” since the term attractiveness may be difficult to grasp in the context of evaluating health constraints. Simpler questioning is also important in light of including participants with diverse backgrounds and without knowledge in health sciences or decision making in future conferences. Reducing complexity should also be considered with regard to the number of judgment decisions asked of the participants.

In contrast to the advantages of applying MCDA in this study, several limitations and areas for future research should be noted in this context. Main issues were uncovered during the weighting procedure and concerned the underlying assumptions of MACBETH. First, participants intuitively expressed dependence between the dimensions of the SF-6D in the weighting process. This fact calls into question the additive form of the utility function in the applied MACBETH approach. While this problem was anticipated, it was disregarded to simplify methodology and allow the application of the MACBETH approach for scoring and weighting without making adjustments. Potential issues connected to the application of MAUT for the valuation of the SF-6D have also been pointed out previously [16]. To address the matter of interactions between dimensions, alternative forms of the value function should be explored. Additional deviations from the original approach could reduce the refusal of making trade-offs by less explicit references to trade-offs within the valuation tasks.

Finally, the methodology applied in this pilot study has restrictions connected to the scaling procedure. Resolving these shortcomings is essential to derive an applicable value set for HRQOL. For simplification, a uniform scaling from 0 to 1 was chosen for the dimensions. This was done by setting the reference values to 0 for the worst performance level and 1 for the best performance level. While the maximum of a value set should be rescaled to a value of 1 to represent perfect health, the allocation of the state death was omitted in the applied procedure. To allow for an adequate valuation of health states considered to be worse than death with negative values, suitable allocation of this state has to be included in future valuation studies.

### Deliberation

Particularly in the small group setting, the deliberative procedure was productive and well received by participants. In addition to providing a setting to consider and discuss judgment decisions, deliberation revealed additional points to consider in the valuation of HRQOL. First, it illustrated the importance of providing sufficient information in an appropriate form and scope. In the conference, the information material provided was concise and thought out. Participants also had the chance to ask and discuss questions when they arose. Especially the latter is not given in survey-based valuations. Nevertheless, the evaluation showed that additional information during the decision process may be necessary. Second, the deliberative procedure illustrated the relevance of ethical aspects in eliciting value sets for HRQOL measures. These considerations are not made explicit in traditional approaches. In the pilot setting, making health-related decisions from a public perspective despite ethical concerns about trade-offs and rationing was challenging for participants. In this context, it is important to note the group composition and distinct characteristics of the pilot study participants. Their background in decision making and public health may have biased the results by both facilitating deliberation and accentuating ethical issues. While the direction of impact is uncertain, this feature of the pilot study emphasizes the potential of bias and the need for including diverse perspectives in deliberative procedures. Overall, the explicit consideration of ethical concerns is an important area of future research for HRQOL valuation in general and consensus-based approaches in particular. The importance of adequately informing participants and addressing potential ethical concerns related to coverage decisions demonstrate the validity of a deliberative valuation procedure.

The approach applied in this study also highlighted the importance of how the process of deliberation is constructed. The procedure applied to validate the expert group results in the plenary assembly demonstrated that a balance between validating and respecting deliberated results is necessary. Further issues of the pilot deliberation were the group size of the plenary assembly and the one-day structure of the conference. The disadvantages of this setting were highlighted in the last plenary session in which participants were confronted with a challenging task and fatigue. While not a focus of the pilot study, future conferences should also implement diverse groups of participants. Overall, further developments of the deliberative process should incorporate organized consensus finding methods to support the group in decision making. In that way, some of the particular challenges of reaching a consensus on HRQOL valuation can be addressed.

Finally, the findings of this pilot study have to be seen in the context of this study being the first attempt in combining MCDA and deliberation to derive a consented value set from a public perspective. The presented results and limitations make it necessary to first improve the methodological approach before conclusions on the feasibility of the concept of a consented value set for HRQOL can be drawn. Further research will address the identified challenges and explore adjusting established valuation techniques for deliberative processes.

## Conclusions

This pilot presents a novel approach for deriving consented value sets for health-related quality of life. However, further research is needed on deliberative processes that yield quantifiable results. Overall, the combination of deliberation and MCDA could address existing issues with current valuation techniques by providing accountable value sets from a public perspective. Especially the successful deliberations of the small groups resulting in consented scores for all SF-6D dimensions indicates the potential of this valuation approach.

This pilot study reveals potential procedural and methodological improvements. First, future conferences should implement smaller group sizes, longer time frames and consensus building techniques. Furthermore, alternatives to the additive value function taking into account interactions between health dimensions and possibilities for reducing the complexity for participants should be investigated. Areas for further research therefore include the adoption of alternative, established valuation techniques for public deliberation in a conference setting. The results of this study also demonstrate the challenges connected to reaching consensus and the importance of ethical considerations in the valuation of health-related quality of life.

## Additional files

Additional file 1: Guidelines for interviews with moderators and software operators. Interview guidelines translated from German to English. (PDF 83 kb)

Additional file 2: Answers to the closed questions of the evaluative questionnaire. Results of questionnaire given to conference participants translated from German to English. (PDF 95 kb)

Additional file 3: Complete coding frame with number of codings in each subcategory. Coding frame used for the qualitative content analysis translated from German to English. (PDF 85 kb)

## Data Availability

All data analyzed during this study are either included in this published article and its supplementary information files or available from the corresponding author on reasonable request.

## Abbreviations

AHP: Analytic Hierarchy Process
HRQOL: Health-related quality of life
MACBETH: Measuring Attractiveness by a Categorical Based Evaluation Technique
MAUT: Multi-attribute utility theory
MCDA: Multi-criteria decision analysis
Q&A: Question and answer
QALYs: Quality-adjusted life-years
QCA: Qualitative content analysis
SF-6D: Short-Form Six-Dimension
