# Supplementary material for: MCDA-based deliberation to value health states: lessons learned from a pilot study
Source: Health Qual Life Outcomes. 2019 Jul 1;17:112. doi: 10.1186/s12955-019-1189-7 (PMC6604444; doi:10.1186/s12955-019-1189-7)
Supplement: Supplementary file 1 — Guidelines for interviews with moderators and software operators. Interview guidelines translated from German to English. (PDF 83 kb) [file 12955_2019_1189_MOESM1_ESM.pdf]

Additional file 1 Guidelines for interviews with moderators and software operators[1]

Notes before beginning

- Thanks for participating
- Background of interview: summarize reasoning for decisions made in small group and evaluation of scoring (and weighting in one interview)
- Procedure: interview around 20 minutes, starts with short questionnaire, insights as moderator and operator important, there are no wrong answers, notes are taken to allow comprehension questions at the end of each topic
- Confidentiality and data protection: get verbal consent for audio recording (show recorder!), confidential handling of data, transcription of interviews
- Let participants fill in questionnaire (information on gender, age, Bachelor's and Master's program, semester, experience with moderation and role in conference)

Interview questions

	**Topic and leading questions**	**Check – Was this mentioned?**<br>***only ask if not volunteered***	**Specific questions**<br>***ask when appropriate***	**Notes**<br>***questions to guide and uphold conversation***
**1)**	**Reasons for evaluations in scoring**<br>In front of you, you can find the scoring results of your group as well as the transcript of your presentation in the plenary assembly. Describe and justify the distances on the scale.	• Definition for restrictions (for a self-determined life)<br>• Reasons of participants<br>→ See summary for this group	• Why is distance X particularly large or small?	• Don't interrupt interviewee<br>• Ask comprehension questions only at the end of each topic<br>• Nonverbal upkeep ("hmm"; "aha"; signal listening by nodding)<br>• Do you have an example for that?<br>• Do you think … plays a role here?<br>• How about … ?
**2)**	**Procedure of discussion in small group**<br>2.1. Please describe, how your group deliberated.<br>2.2. Which difficulties of the participants did you notice?	• Reference to "good deliberation":<br>o Did everyone get a chance to speak?<br>o Were opinions suppressed?<br>o Was discussion influenced by individuals?	• Did you notice anything that may have biased the results?	• Don't interrupt interviewee<br>• Ask comprehension questions only at the end of each topic<br>• Nonverbal upkeep<br>• Do you have an example for that?<br>• Do you think … plays a role here?<br>• How about … ?

**3) Scoring procedure in plenary assembly**<br>3.1. How did you perceive the presentation and discussion of your group's results in the plenary assembly?<br>3.2. How would you assess the deliberation in the plenary assembly?	• Did group result come across in plenary assembly?<br>• Impression of discussion in plenary assembly<br>• Reference to "good deliberation":<br>o Did everyone get a chance to speak?<br>o Were opinions suppressed?<br>o Was discussion influenced by individuals?	• Which causes would you identify?	• Don't interrupt interviewee<br>• Ask comprehension questions only at the end of each topic<br>• Nonverbal upkeep<br>• Do you have an example for that?<br>• Do you think … plays a role here?<br>• How about … ?
**4)∗ Weighting procedure in plenary assembly**<br>4.1. How would you describe the process of deliberation in the plenary assembly?<br>4.2. Which difficulties of the participants did you notice?<br>4.3. In the end, the participants decided not to agree on a quantitative result. From your point of view, what were the main reasons for not achieving a consented result for the weighting procedure?	see 2) and 3)		• Don't interrupt interviewee<br>• Ask comprehension questions only at the end of each topic<br>• Nonverbal upkeep<br>• Do you have an example for that?<br>• Do you think … plays a role here?<br>• How about … ?

∗ *These questions are only for the moderator and software operator of the weighting session.*

Manual

- Scheduled duration of interviews: 15 to 20 min (+15 min for interview on weighting); keep track of time for each topic!
- Get consent for audio recording, show recorder and place so that not visible.
- Pay attention to notes before beginning.
- Have participants fill in questionnaire before the interview.
- Start recording.
- Don't interrupt interviewee and ask comprehension questions only at the end of each topic.
- After turning off recording, stay attentive (participants could add something in relaxed atmosphere → document in postscript).
- Make notes in postscript immediately after interview.

[1]*These interview guidelines were translated from the original German version to English. The interviews were conducted in German.*
