# Supplementary material for: MCDA-based deliberation to value health states: lessons learned from a pilot study
Source: Health Qual Life Outcomes. 2019 Jul 1;17:112. doi: 10.1186/s12955-019-1189-7 (PMC6604444; doi:10.1186/s12955-019-1189-7)
Supplement: Supplementary file 2 — Answers to the closed questions of the evaluative questionnaire. Results of questionnaire given to conference participants translated from German to English. (PDF 95 kb) [file 12955_2019_1189_MOESM2_ESM.pdf]

Additional file 2 Answers to the closed questions of the evaluative questionnaire

Additional table 1 Results of the evaluative questions about the conference procedure

	Strongly disagree		Disagree		Neutral		Agree		Strongly agree	
	n	%	n	%	n	%	n	%	n	%
**I was able to introduce my perspective into the discussion.**	0	0.0 %	1	4.2 %	4	16.7 %	9	37.5 %	10	41.7 %
**Participants responded to statements of others.**	0	0.0 %	0	0.0 %	5	20.8 %	13	54.2 %	6	25.0 %
**Participants interrupted each other often.**	11	45.8 %	11	45.8 %	2	8.3 %	0	0.0 %	0	0.0 %
**Participants constructively worked towards achieving consensus.**	0	0.0 %	3	12.5 %	4	16.7 %	13	54.2 %	4	16.7 %
**I am satisfied with the result achieved in the small group session.**	0	0.0 %	0	0.0 %	2	8.3 %	3	12.5 %	19	79.2 %
**I am satisfied with the result achieved in the plenary session.**	1	4.2 %	11	45.8 %	4	16.7 %	6	25.0 %	2	8.3 %
**The provided information was understandable and helpful.**	0	0.0 %	1	4.2 %	4	16.7 %	7	29.2 %	12	50.0 %
**After the introduction, content and objectives of the conference were clear to me.**	0	0.0 %	3	12.5 %	1	4.2 %	11	45.8 %	9	37.5 %
**There was sufficient room for questions.**	1	4.2 %	0	0.0 %	0	0.0 %	6	25.0 %	17	70.8 %

Additional table 2 Results of the questions on the Perspective Taking Scale

	Does not describe me well		Rather does not describe me well		Neutral		Describes me rather well		Describes me well	
	n	%	n	%	n	%	n	%	n	%
**Before criticizing somebody, I try to imagine how I would feel if I were in their place.**	0	0.0 %	1	4.2 %	8	33.3 %	5	20.8 %	10	41.7 %
**If I'm sure I'm right about something, I don't waste much time listening to other people's arguments.**	3	12.5 %	12	50.0 %	4	16.7 %	5	20.8 %	0	0.0 %
**I sometimes try to understand my fellows better by imagining how things look from their perspective.**	0	0.0 %	3	12.5 %	2	8.3 %	10	41.7 %	9	37.5 %
**I believe that there are two sides to every question and try to look at them both.**	1	4.2 %	2	8.3 %	4	16.7 %	12	50.0 %	5	20.8 %
**I sometimes find it difficult to seethings from the "other guy's" point of view.**	2	8.3 %	8	33.3 %	5	20.8 %	6	25.0 %	3	12.5 %
**I try to look at everybody's side of a disagreement before I make a decision.**	0	0.0 %	3	12.5 %	1	4.2 %	9	37.5 %	11	45.8 %
**When I'm upset at someone, I usually try to "put myself in his shoes" for a while.**	1	4.2 %	7	29.2 %	8	33.3 %	7	29.2 %	1	4.2 %
