# Supplementary material for: MCDA-based deliberation to value health states: lessons learned from a pilot study
Source: Health Qual Life Outcomes. 2019 Jul 1;17:112. doi: 10.1186/s12955-019-1189-7 (PMC6604444; doi:10.1186/s12955-019-1189-7)
Supplement: Supplementary file 3 — Complete coding frame with number of codings in each subcategory. Coding frame used for the qualitative content analysis translated from German to English. (PDF 85 kb) [file 12955_2019_1189_MOESM3_ESM.pdf]

Additional file 3 Complete coding frame with number of codings in each subcategory

**1 Aspects of procedure evaluated**	
1.1 Exhaustion or excessive demand	14
1.2 Insecurity about procedure	9
1.3 Insecurity about topic	5
1.4 Size of plenary assembly	8
1.5 Time management	8
1.6 Organizational issues	8
1.7 Questioning work of small groups	13
1.8 Missing information	10
1.9 Other	6
**2 Aspects of methodology evaluated**	
2.1 Understanding	15
2.2 Independence of dimensions	6
2.3 Trade-off between dimensions	9
2.4 Abstraction from personal level	7
2.5 Finding consensus	10
2.6 Unclear definitions	29
2.7 Other	4
**3 Aspects of participants' behavior evaluated**	
3.1 Conversational atmosphere	17
3.2 Interaction	13
3.3 Balanced participation	3
3.4 Unbalanced participation	17
3.5 Willingness to find consensus	17
3.6 Unwillingness to find consensus	10
3.7 Moderation	6
3.8 Other	6
**4 Aspects of research approach evaluated**	
4.1 Complexity of research question	8
4.2 Assumptions and implementation	6
4.3 Influencing results	8
4.4 Ethical concerns	11
4.5 Ethical acceptability	3
4.6 Practical application	6
4.7 Other	0
**5 Suggestions for improvement**	
5.1 Information for participants	6
5.2 Approach for weighting	3

5.3 Procedure for scoring	15
5.4 Group organization	4
5.5 Time management	3
5.6 Other	1

6 Reasons for evaluation of difference in attractiveness

6.1 Intuition	5
6.2 Expectations of society	9
6.3 Self-expectation	10
6.4 Satisfaction	10
6.5 Tolerance or Adjustment	15
6.6 Marginal benefit	21
6.7 Level of impairment	23
6.8 Needs	5
6.9 Autonomy or self-determination	23
6.10 Evaluation of an example	18
6.11 Unclear	8
6.12 Other reasons	4

7 Dimensions which should have greater weight

7.1 None	8
7.2 Physical functioning	1
7.3 Role limitation	1
7.4 Social functioning	0
7.5 Pain	5
7.6 Mental Health	4
7.7 Vitality	0

8 Dimensions which should have smaller weight

8.1 Physical functioning	1
8.2 Role limitation	1
8.3 Social functioning	0
8.4 Pain	0
8.5 Mental Health	0
8.6 Vitality	3

9 Difference in attractiveness

9.1 No difference	1
9.2 Very weak	4
9.3 Weak	4
9.4 Moderate	3
9.5 Strong	2
9.6 Very strong	8
9.7 Extreme	4
